# Co-Production at the Strategic Level: Co-Designing an Integrated Care System with Lay Partners in North West London, England

**DOI:** 10.5334/ijic.2470

**Published:** 2016-05-03

**Authors:** Michael Morton, Elisabeth Paice

**Affiliations:** Co-Chair of Lay Partners Advisory Group, North West London Whole System Integrated Care Pioneer, GB; Visiting professor, Imperial College London, GB; Co-Chair of Lay Partners Advisory Group, North West London Whole System Integrated Care Pioneer, GB

**Keywords:** Coproduction, Co-design, Integrated care, Lay Representatives, Patients, Citizens

## Abstract

In North West London, health and social care leaders decided to design a system of integrated care with the aim of improving the quality of care and supporting people to maintain independence and participation in their community. Patients and carers, known as ‘lay partners,’ were to be equal partners in co-production of the system.

Lay partners were recruited by sending a role profile to health, social care and voluntary organisations and requesting nominations. They formed a Lay Partners Advisory Group from which pairs were allocated to system design workstreams, such as which population to focus on, financial flow, information technology and governance. A larger and more diverse Lay Partners Forum provided feedback on the emerging plans.

A key outcome of this approach was the development of an integration toolkit co-designed with lay partners. Lay partners provided challenge, encouraged innovation, improved communication, and held the actions of other partners to account to ensure the vision and aims of the emerging integrated care system were met.

Key lessons from the North West London experience for effective co-production include: recruiting patients and carers with experience of strategic work; commitment to the vision; willingness to challenge and to listen; strong connections within the community being served; and enough time to do the work. Including lay partners in co-design from the start, and at every level, was important. Agreeing the principles of working together, providing support and continuously recruiting lay representatives to represent their communities are keys to effective co-production.

## Introduction

In 2013 North West London’s clinical commissioners (organisations that plan and purchase care to improve the health of defined populations), primary and secondary health care providers, local authorities and the voluntary sector came together to plan ‘whole system integrated care.’ The aim was to improve the quality of care, empowering and supporting people to maintain independence and to lead full lives as active participants in their community. Partners felt if the vision of integrated care was to be achieved, it had to be a social movement led by people who use services, their families, carers and the public; supported by staff at every level in every care setting [1]. To this end, patients, carers and other service users (called ‘lay partners’) were to be equal partners in the design and delivery of the new system.

Three levels of co-production were relevant to this work:

1. Individual level. The patient (and their carer where relevant) working in partnership with professionals to design and direct their own care.
2. Service level. People who use a specific service (eg disease-specific care) working with commissioners and providers of that service.
3. Strategic level. People with experience of using services, and of working at a policy-making level in any field, working with professionals to design a system of care.

The programme was in two phases: a central design phase (2013–2014); and an ‘early adopter’ implementation phase (2015–2016). In this paper we will focus mainly on the design phase.

## Co-production at strategic level

“Previously as a patient and community member I was always struck, and often frustrated, by the fact that so much had been decided at a strategic level before we were involved, thus restricting what we could subsequently influence.” Lay partner

**Figure 1:**
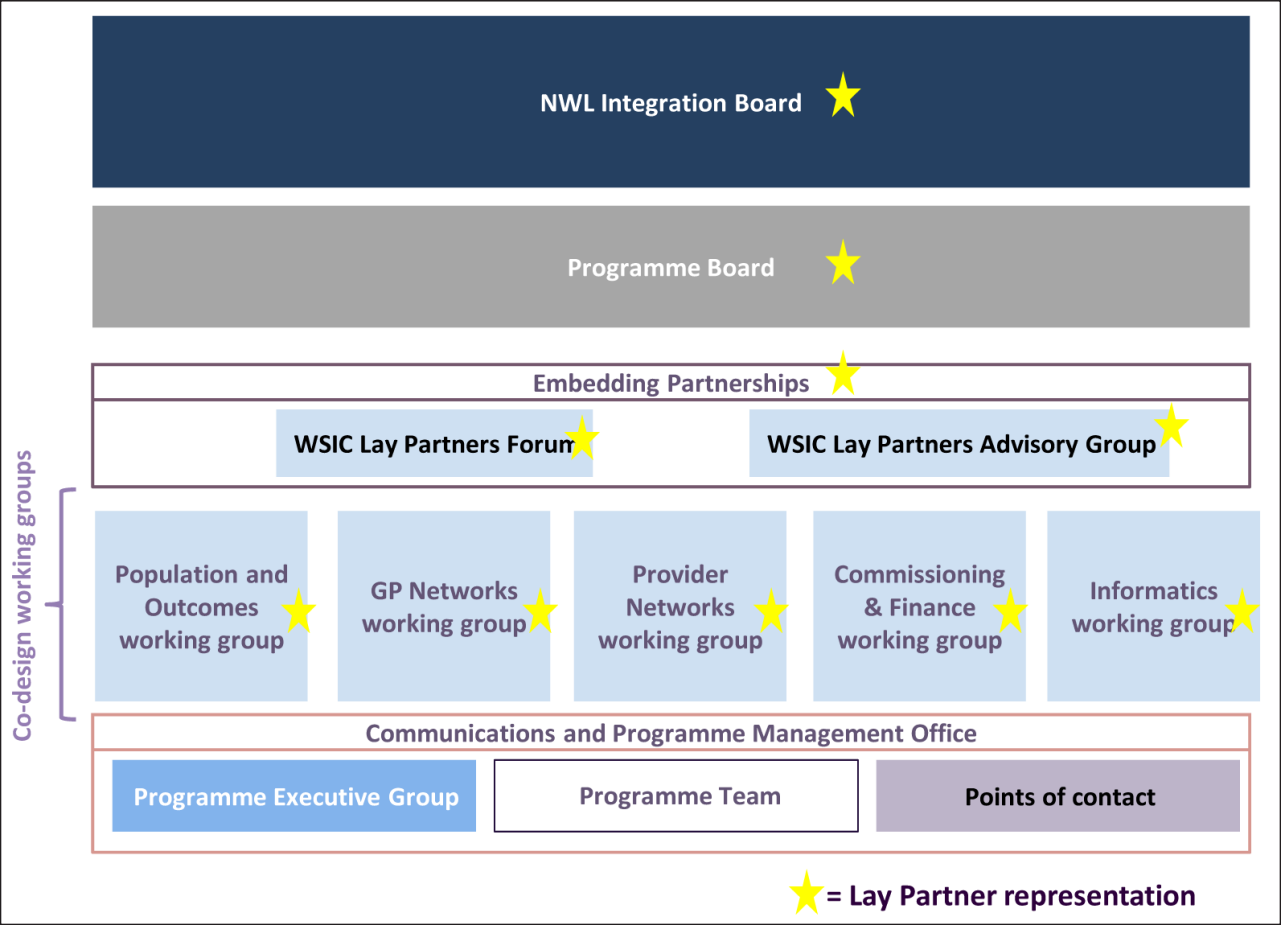
Governance structure for Whole System Integrated Care: design phase.

The design phase was driven by five working groups, meeting fortnightly, each chaired by a senior doctor and including a range of senior clinicians and managers from the partner organisations. An overarching group, called “Embedding Partnerships”, governed the lay partner involvement. Embedding Partnerships consisted of a Lay Partners Advisory Group with 15–25 people and a Lay Partners Forum with around 100 people (Fig. 1). The Lay Partner Advisory Group sent pairs of representatives to each of the five working groups, where they were full members.

## Recruiting and selection

The first task was to find lay partners with the time, ability and commitment to work at a strategic level, at the pace necessary to achieve the programme’s aims. A ‘role profile’ set out what lay partners would be expected to do, how many meetings they would need to attend and that the role was unpaid. The role profile was sent to directors of social care, general practices, local charities, and other organisations, asking for nominations from among their patient or carer groups. Nominees were invited to a workshop at which the vision of ‘whole systems integrated care’ was explained and participants were encouraged to consider what attributes a lay partner would require.

Forty people attended the event, 25 expressed an interest in going forward and 15 became the active core of the Lay Partner Advisory Group. Those who withdrew cited the time commitment and the lack of remuneration. As the programme got underway, further nominations were encouraged to replace people leaving. Additional members were selected on the basis of the diversity they brought to the group.

At the same time a wider group of patients and carers, including those without time to meet frequently or without experience of working at a strategic level, were recruited to join a Lay Partners Forum of 100 or so. We employed an expert in patient and public engagement to seek out people from under-represented communities and more diverse backgrounds. At the first meeting of the Forum we consulted participants about what would make the meetings work well for them, and agreed where and when they should be held. They emphasised the need for good communication, being valued and listened to, and being able to see the results of their contribution.

‘Embedding Partnerships’ was initially chaired by one of the authors (EP), a retired doctor. Subsequently the other author (MM), a lay partner, was elected a co-chair by the membership.

## Working together

Some professionals were uncomfortable at first with the concept of co-production at a strategic level. Negative comments included that: lay partners would not be able to grasp the complexity; they would slow things down; they would not understand that resources were limited; and that the work was at too early a stage to benefit from their input.

To address these arguments, we made sure lay partners were available to attend each of the working groups from the first meeting, so that they were part of the team from the start. The Lay Partners Advisory Group met fortnightly, and provided an opportunity for the programme team to brief and support the members and for members to share their experiences and seek each other’s opinions, so that they felt more confident at challenging the professionals where necessary. The Group developed a ‘touchstone’ of principles of co-production [2,3], based on the work of Nesta [4]. Some examples of the principles are as follows: Regardless of professional position or lived experience – we come to the table as equal partners; we strive to remove paternalism and keep our written communications plain and simple – avoiding jargon; and our thinking is always person-centred – putting the person not the system at the heart of the process.

These touchstone principles were adopted by the whole programme and influenced the behaviours of all partners. Gradually the culture changed and lay partners were seen as essential to the work. They brought experiences and skills from their varied working lives and an understanding of the communities that they lived in. They were less risk-averse than professionals, encouraging ambition and innovation. Their presence encouraged better behaviour among professionals. They were represented at every level in the programme, often taking the role of chair or co-chair. They were in demand as speakers, as panellists for selection of early adopter sites and as leaders in local implementation of integrated care.

“We’ve found that the patient perspective can often help bring about shared understanding where professionals with different opinions may have disagreed. In some cases we acted as arbitrators of these different viewpoints.” Lay partner

The Lay Partners Forum met quarterly and was an opportunity for the programme team to listen to feedback, share ideas and discuss progress with a wider and more diverse group of lay partners.

## Outcome

By the end of the first year, the programme had co-produced the “North West London Integration Toolkit”, which provided the design principles for implementation at early adopter sites across the region [1]. The defining contribution made by lay partners to this work was recognised in a government report [5] an independent evaluation [6] and an NHS award for Patient Champion of the Year [7]. The next challenge is to ensure that the culture of co-production is sustained as the programme of implementation through local early adopters gets underway.

## Lessons learned

Recruit the right people. The most effective and committed lay partners had these qualities:

- Lived experience as a patient and / or carer.
- Experience of working at a strategic level, in any field.
- Commitment to a shared vision - not single issue champions.
- Willingness to challenge professionals
- Ability to listen, weigh arguments and help reach a consensus.
- Diverse connections within the community.
- Time to read papers, attend meetings and communicate.

Provide support. It was important that a core group of committed lay partners stayed in the Group from the start, gaining the trust of professionals and helping to embed a culture of co-production. We were fortunate in recruiting lay partners who were willing to give so much of their time over such a long period. It was important that they had support from the leadership and programme management and that they were valued and respected. We could have done more to support those who could not take part because of other responsibilities, eg by offering remuneration, holding evening meetings, or supporting carers.

“I remain involved because the programme is purposeful, insightful and really committed to making change, we work well as a team of very different but complementary interests and experiences, I see the fruits of our efforts and I feel motivated by what I believe is a shift in culture in the programme to really hear and understand the value of what patients, carers and families have to say at the highest level of decision making.” Lay partner

Continued recruitment. The need for lay partners extended beyond the design phase, as early adopters began local implementation. Those who had been on the journey from the start were much in demand. In retrospect we should have been bringing more new people in throughout the design phase to meet this demand.

## Conclusions

In summary, we found that co-production at a strategic level added value to the design of our integrated care system, and embedded a culture of patient-centred working that we might not otherwise have achieved.

## Competing Interests

The authors declare that they have no competing interests.

## Author’s Information

Michael Morton is a retired businessman with international experience, living with long-term conditions. He has been involved in the NHS as a representative patient for the past 8 years, with particular interest, since 2011, in the developing integrated care programme and its potential benefits for the provision of care for patients.

Elisabeth Paice was previously a consultant rheumatologist and the postgraduate dean for London. From 2011 she has been a leader in North West London’s integrated care programme, chairing the initial pilots and leading on patient engagement.
